# The discrimination of interaural level difference sensitivity functions: development of a taxonomic data template for modelling

**DOI:** 10.1186/1471-2202-14-114

**Published:** 2013-10-07

**Authors:** Balemir Uragun, Ramesh Rajan

**Affiliations:** 1 Physiology Department, Monash University, Clayton, Victoria 3800, Australia

## Abstract

**Background:**

A major cue for the position of a high-frequency sound source in azimuth is the difference in sound pressure levels in the two ears, Interaural Level Differences (ILDs), as a sound is presented from different positions around the head. This study aims to use data classification techniques to build a descriptive model of electro-physiologically determined neuronal sensitivity functions for ILDs. The ILDs were recorded from neurons in the central nucleus of the Inferior Colliculus (ICc), an obligatory midbrain auditory relay nucleus. The majority of ICc neurons (~ 85%) show sensitivity to ILDs but with a variety of different forms that are often difficult to unambiguously separate into different information-bearing types. Thus, this division is often based on laboratory-specific and relatively subjective criteria. Given the subjectivity and non-uniformity of ILD classification methods in use, we examined if objective data classification techniques for this purpose. Our key objectives were to determine if we could find an analytical method (A) to validate the presence of four typical ILD sensitivity functions as is commonly assumed in the field, and (B) whether this method produced classifications that mapped on to the physiologically observed results.

**Methods:**

The three-step data classification procedure forms the basic methodology of this manuscript. In this three-step procedure, several data normalization techniques were first tested to select a suitable normalization technique to our data. This was then followed by PCA to reduce data dimensionality without losing the core characteristics of the data. Finally Cluster Analysis technique was applied to determine the number of clustered data with the aid of the CCC and Inconsistency Coefficient values.

**Results:**

The outcome of a three-step analytical data classification process was the identification of seven distinctive forms of ILD functions. These seven ILD function classes were found to map to the four “known” ideal ILD sensitivity function types, namely: Sigmoidal-EI, Sigmoidal-IE, Peaked, and Insensitive, ILD functions, and variations within these classes. This indicates that these seven templates can be utilized in future modelling studies.

**Conclusions:**

We developed a taxonomy of ILD sensitivity functions using a methodological data classification approach. The number and types of generic ILD function patterns found with this method mapped well on to our electrophysiologically determined ILD sensitivity functions. While a larger data set of the latter functions may bring a more robust outcome, this good mapping is encouraging in providing a principled method for classifying such data sets, and could be well extended to other such neuronal sensitivity functions, such as contrast tuning in vision.

## Background

The ability to identify the location of a sound source is a core auditory ability for many daily purposes [1]. Our ability to accurately localize sounds depends on coding, by neurons in the Central Nervous System, of various cues to the location of the sounds. For on-going high frequency sounds, the major cue for azimuthal location of the sound source is the difference in intensity/level (formerly Interaural Intensity Differences, now Interaural Level Differences; IIDs/ILDs) [2]. ILDs are the difference in sound levels at the two ears as a sound source moves about an animal and are created by head and body shadowing effects which affect high frequency sounds more than low frequency sounds [3]. There is a vast literature on the importance of ILDs and how neurons at various brain levels respond to ILDs that cover a wide azimuthal range across frontal space, from opposite one ear across to opposite the other. In mammals this cue is first functionally coded by neurons in the auditory brainstem, and then relayed to the Inferior Colliculus (IC), but it is clear that in some species at least (including the rat studied here), ILD sensitivity is also created *de novo* in many IC neurons [4].

Different IC neurons appear to use different combinations of interactions between excitatory and inhibitory inputs to code ILDs (a set of neuronal operations that also appears to be used in auditory cortex), [5] producing a diversity of forms of ILD sensitivity in neurons in the one auditory structure; this diversity argues against using a single network model to describe all the different forms of ILD sensitivity.

### Introduction to data normalization

Data normalization is a scaling process for numbers in a data array and is used where a great heterogeneity in the numbers renders difficult any standard statistical analysis. The data is often normalized before any application process and therefore data normalization is usually termed as data pre-processing. Many different data normalization techniques have been developed in diverse scientific fields, e.g. in statistical analysis for applications such as in diagnostic circuits in electronics [6], temporal coding in vision [7], predictive control systems in seismic activities [8], modeling Auditory Nerve stochastic properties [9], modeling labor market activity [10], pattern recognition [11], and most extensively in microarray data analysis in genetics, [12-20].

The need for data normalization is determined by the user and depends on the application. Thus the purpose of data normalization depends on the proposed application, and includes use of linear scaling to compress a large dynamic range [6], scaling of values to correct for variation in laser intensity [18], handling obscure variation [12] or removing systematic errors in data [11,15,17,20], or efficiently removing redundancy in a non-linear model as an optimal transformation for temporal processing [7]. Although the benefits of data normalization depend on data type, data size and normalization method (which can vary between different fields), generally the advantages of data normalization are (a) to give a more meaningful range of scaled numbers for use, (b) to rearrange the data array in a more regular distribution, (c) to enhance the correctness of the subsequent calculations, and (d) to increase the significance or importance of the most descriptive numbers in a non-normally distributed data set.

### Introduction to data dimension reduction technique

Principal component analysis (PCA) is a statistical tool to reduce the dimensions of a large data set for the purpose of data classification when a data set can be described in a number of different ways, or described by a number of different variables (such as slope steepness, cut-off position, peak location, maximum firing rate etc.), and is therefore said to possess many dimensions. Such data becomes difficult to classify because it is often not known which of these dimensions are the most important or, indeed, if only one of them is the most important. In such a case, some means has to be devised in order to reduce the dimensions in the data set to a single dimension across all the data. This single dimension can then be used to differentiate between sub-groups within the overall data set. PCA is a powerful statistical tool that does precisely this.

The PCA is used as an independent statistical method for data classification to handle both metric and multivariable types of data [21]. In the PCA, the data variables are largely dependent on one another; in fact, if data were not correlated then principal components would not be suitable for data dimension reduction. *Barlett’s Sphericity Test* can be used to verify the appropriate conditions for the data [22], but the details of this test are beyond the scope of this manuscript.

### Introduction to cluster analysis

Data classification is a way of segregating similar types of data groups into homogenous clusters. Each of these compact data groups contains a number of data-elements with comparable characteristics. In data classification studies, two methods are generally used to distinguish the classified data, namely: *Supervised* (discriminant analysis) and *Unsupervised* (data clustering) classification [23].

Data characterization can be planned as a two-step procedure consisting of the combination of PCA for reduction of data dimensions followed by *Cluster Analysis* for grouping similar types of data objects. This technique has been widely used in several different types of applications in a diverse range of scientific fields including in crime analysis [24], in finding the relationship between retention parameters and physiochemical parameters of barbiturates [25], in chemo-metric methods in characterizing steel alloy samples [26], in drug design [27], in isolating single unit activities for data acquisition [28], and in microarray based gene identification [29,30]. This combined technique has been reviewed by [31] for several clustering algorithms, and they have emphasized the importance of applying PCA prior to Cluster Analysis for high dimensional data.

## Results

**Figure 1:**
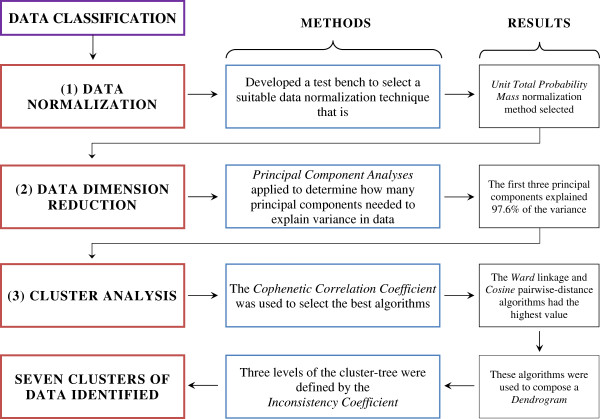
**The data classification procedure.** The three step data classification process resulted with seven ILD sensitivity functions data groups. In this three-step process; (1^st^) several data normalization techniques tested for our data and the UTPM (unit total probability mass) data normalization method was most suitable one, (2^nd^) first three principal components of PCA were selected and these were good enough to present entire data by the 97.6 % variance explained, (3^rd^) Cluster Analysis based on the Ward linkage and Cosine pairwise-distance algorithms those selected algorithms helped to compose a dendrogram where Inconsistency Coefficient determines the number of clustered data.

In this section, we used a three-step analytical data classification process to produce the results, and these steps are: (1) data normalization, (2) data dimension reduction and (3) cluster analysis, as all shown in Figure 1.

### Data normalization: results

In general terms, normalization is simply signal intensity divided by a reference value, and serves to reduce systematic errors in data variables [32]. Data normalization also maximizes variance [22], which is especially important before applying the data dimension reduction technique for ILD type data of this study. Data normalization is sometimes a prerequisite for data analysis in statistics, and finding a suitable scaling technique for the data is therefore an important task.

Since the appropriate normalization technique for ILD data is unknown, we chose to test seven different techniques against our library of nine ideal ILD function variants. These seven data-normalization techniques have been widely used in many different applications but also in similar types (multivariate) of data. These are namely data normalization by *mean correction,* by *a maximum value,* by *each vector’s maximum value,* by *each vector’s standard deviation, data standardization, Logarithmic normalization* and*, unit total probability mass* (UTPM) as detailed in [33].

**Figure 2:**
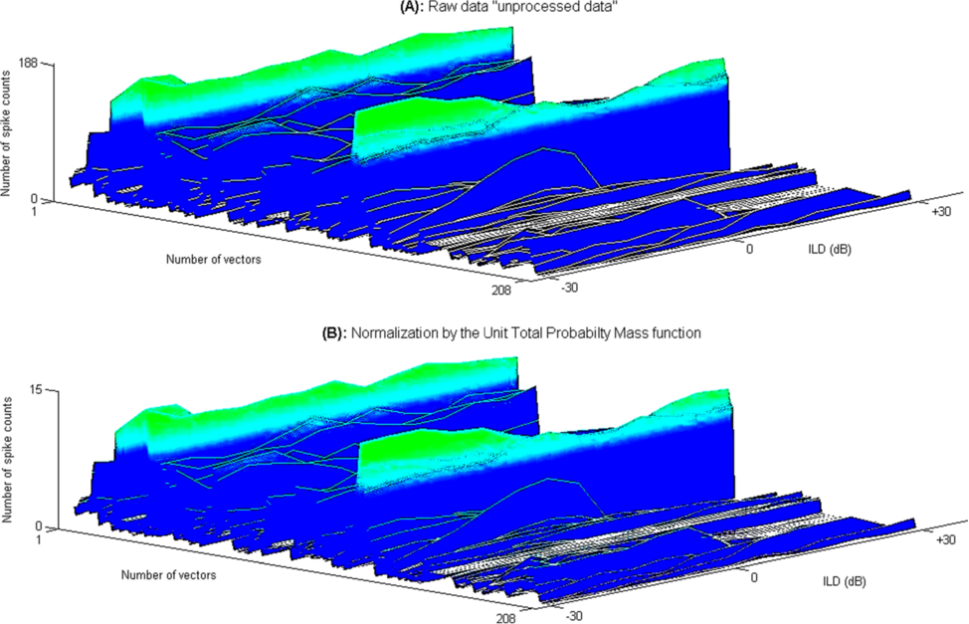
**The raw and normalized data compared.** The raw as unprocessed data consists of 208 vectors, and each vector (panels) has got varying number of spike counts between zero and 188 for 13 ILD levels from −30 dB to +30 dB with the increment of 5 dB **(A)**, and (Additional file 1). The normalized data by the UTPM function perfectly preserves the shapes of raw-data while scales the number of spike counts down by %92.38 **(B)**, Table 1. Therefore, there is not much differences observed between normalized data in **(B)** and raw data in **(A)**.

We applied these seven normalization techniques to the ILD data to see which could best reduce the variance in the neuronal spike counts obtained in the electrophysiological recordings. The result of applying all seven data normalization techniques in this normalization “test bench” is tabulated in the Table 1 along with the quantitative conclusion of the analysis using each normalization technique. It is evident that the best method for normalization was the UTPM data normalization technique. The data normalized by the UTPM function perfectly preserves the shapes of raw-data while it scales the number of spike counts down by ~%90. Hence there are not many differences observed between this normalized data (Figure 2B) and the raw data (Figure 2A). This normalization technique was therefore applied before exploring the ILD data with PCA and Cluster Analysis.

**Table 1:** The nine prototypical ILD functions

**Seven data normalization methods**	**The nine prototypical ILD functions (min: 0.0 and max: 100, with ±6 %)**								
	**(A)**	**(B)**	**(C)**	**(D)**	**(E)**	**(F)**	**(G)**	**(H)**	**(I)**
**(1)** Vn (i, j) = *X*_*n*_(*i*, *j*) − *μ*_*n*_	-46/49	-67/68	-69/48	-37/59	-23/73	-43/71	-64/60	-42/46	-5.7/8
**(2)** Vn (i, j) = *X*_*n*_(*i*, *j*)/*max*{*max*{*X*_*n*_(*i*, *j*)}}	0.01/1	0.01/1	0.01/1	0.01/1	0.01/1	0.01/1	0.01/1	0.01/1	0.01/1
**(3)** Vn (i, j) = *X*_*n*_(*i*, *j*)/*max*{*X*_*n*_(*i*, *j*)}	0.01/1	0.01/1	0.01/1	0.01/1	0.01/1	0.01/1	0.01/1	0.02/1	0.24/1
**(4)** Vn (i, j) = *X*_*n*_(*i*, *j*)/*σ*_*n*_	0.04/2	0.02/2	0.04/2	0.06/2	0.04/3	0.03/3	0.02/2	0.1/23	0.6/51
**(5)** Vn (i, j) = log_2_(X_n_(i, j)) − log_2_(μ_n_)	-4.6/1	-6/1.7	-5.3/1	-4/1.3	-4.3/2	-4.6/2	-4.8/1	-4.4/1	-1/0.7
**(6)** Vn (i, j) = $\frac{X_{n}\left(i,j\right)}{\sum_{n}X_{n}}\cdot \mu_{n}$	0.1/7	0.1/7	0.08/7	0.09/7	0.08/7	0.09/7	0.09/7	0.23/7	0.1/7.
**(7)** Vn (i, j) = $\frac{X_{n}\left(i,j\right)-\mu_{n}}{\sigma_{n}}$	-1.1/1	-1.1/1	-1.8/1	-1/1.7	-0.7/2	-1.3/2	-1.5/1	-1.8/1	-1.4/1

Where, the number of raw and number of columns for the matrix form of normalized *“Vn”* and raw data “*Xn*” are both comprise same “*i*” number of raw, “*j*” number of columns and *“n”* number of ILD patterns. These nine prototypical ILD functions are: (A) Sigmoidals w/varying # of spike counts, (B) Sigmoidals w/varying position of cut-off, (C) Sigmoidals w/varying steepness of the slope, (D) Peaked w/ varying # of spike counts, (E) Peaked w/ varying position of cut-off, (F) Peaked w/ varying steepness of the slope and position of cut off, (G) Peaked w/ unilateral transition to Sigmoidal, (H) Peaked w/ bilateral transition to Intensive, and (I) Intensive w/ varying number of spike counts. The seven data normalization methods are: (1) Mean correction, (2) Overall maximum value, (3) Each vector maximum value, (4) Standard deviation, (5) Logarithmic, (6) Unit total probability mass, and (7) Data standardization. In addition, seven data normalization methods (with the equations) were applied to nine (from “A” to “I”) prototypical ILD functions. The result was presented in this table with the minimum of minima (four vectors)/ maximum of maxima (four vectors) values were all shown in spike counts.

### PCA result

#### PCA for ILD data

**Figure 3:**
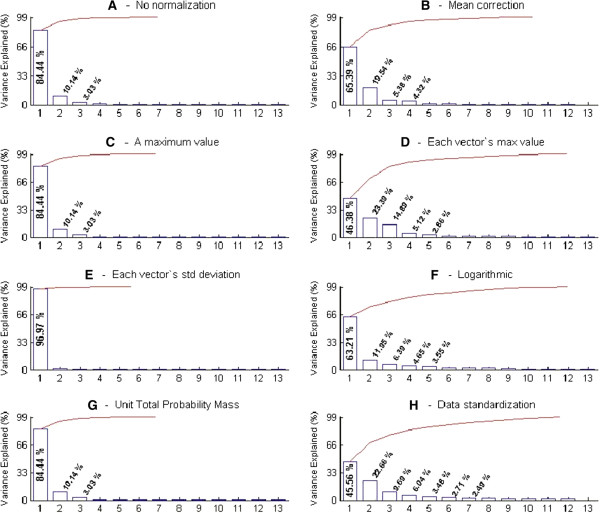
**The Scree-plot used for determining the number of principal components.** The Scree-plot (the lines above the bar plots) and variance explained by the percentage bar plots, are both used for the number of principal component selection towards PCA for seven normalization techniques. Raw **(A)** and seven different normalized data **(B**-**H)** all applied for PCA. In a result, the variances information of each set of principal components (PC1, PC2, PC3 … and PC13) is extracted from the PCA to show the significance. Either higher variance values of principal components, or prior to bending point “elbow” in the Scree-plot, they both indicate necessary number of principal component usage for the reduced data dimension representation.

The normalization test bench analyses detailed above showed that the UTPM data normalization technique appeared to be the most suitable normalization technique to reduce the variance in our electrophysiological data. Nevertheless in the PCA analyses, we conducted PCA on all seven normalization techniques to determine the number of principal components needed to account for the variance in data normalized with each of these normalization techniques (Figure 3), as this is an issue that is critical for data classification below. The results are summarized in Figure 3 which presents, for each normalization data type the number of significant principal components together with the variance explained by those principal components, as shown by the percentage data and the *Scree-plot* in the figure.

Figure 3 PCA confirms that the UTPM data normalization is among the normalization techniques that can be represented by “sufficiently” few principal components (Figures 3A, C, E, G versus Figure 3B, D, F, H). It was not the normalization technique that needed the lowest number of principal components: for the normalization technique using division by each vector’s standard deviation, the first principal component (PC1) was sufficient to explain 96.77% of the variance (Figure 3E). For three other normalization techniques (which included the UTPM data normalization technique), the first three principal components (PC1, PC2, and PC3) were needed to account for a significant amount of the variance and explained 84.44%, 10.14% and 3.03% of the variance respectively (Figures 3A, C and G). The remaining four normalization techniques (Figure 3B, D, H and F) required more than three principal components to represent the variance in the data.

Although the technique of data normalization by each vector’s standard deviation was the most parsimonious in the sense that the PCA can be represented by a single principal component, this normalization technique was not used for our data. Our normalization test-bench had shown (see Table 1) that this normalization technique when applied to our ILD data did not markedly affect the variance in the neuronal spike counts across the ILD functions. Note that this also illustrates the importance of conducting the normalization work-test-bench exercise.

### Result: Finding the right number of principal components

Selection of the correct number of principal components is important for reducing data dimensionality in PCA. The selection of the number of principal components is not an arbitrary task, despite the fact that it is general practice to select the very first few principal components, often only the first two. The first principal component (PC1) is the projection of the given points and it has the maximum variance among all possible linear coordinates. The second Principal Component (PC2) has maximum variance along an axis orthogonal to the first principal component [34]. In usual practice with two-dimensional PCA, the first two principal components allow efficient visual representation of data and there are certainly some specific examples where a small number of principal components appear appropriate to visualize cluttered data distribution. These include the linear combination of gene expression levels on the first three principal components represented in a three-dimensional plot [32], or rotating three-dimensional Principal Components representation for the analysis of tumor karyotypes in [35] or in three-dimensional object recognition application [36], or first two principal components utilized in the neural activity data analysis for the data classification [28]. Thus the PCA technique offers the least information loss when the first few principal components can account for the greatest variance in the data [37].

It must also be noted that if a large number of principal components is needed to represent a data set, then data-normalization is not efficiently applied [38]. This was also observed for our data with several of the normalization techniques we tested (see Figures 3B, D, F and H).

Despite all these advantages of arbitrarily restricting PCA outcomes to the first few principal components, a more efficient and principled approach is to apply some decision process to the selection of the appropriate number of principal components. This can be applied to our data to decide the number of principal components to be used [22]. Similar “decision-process” test procedures for determining the number of principal components to be used have been discussed in other contexts by [21,39] and we list here one set of decision rules that can be applied:

(i) The Scree-plot gives Eigenvalues versus number of principal components. The point of change (the elbow of the curvature) in the figures (Figure 3), which distinguishes the number of principal components, is the highest percentage to be retained.

(ii) *Kaiser’s* rule retains all components with Eigenvalues greater than one [40], and is a way of measuring the common variance of variables.

(iii) *Horn’s* procedure is similar to Kaiser’s Rule; it gives fewer principal components than would Kaiser’s Rule.

(iv) Explained variance is a way of looking for the variance explained by the first few principal components. This may be a sufficient way to decide whether more principal components are required.

We used both the Scree-plot and the total-variance to select the number of principal components for our data. The PCA result for data treated with our preferred normalization method, the UTPM method, is given as a percentage of principal components’ variances using Equation 2, and is represented visually in Figure 3G and values tabulated in Table 2. The data show that for ILD data normalized by this method, the first three principal components appear to account for the greatest amount of variance.

**Table 2:** Thirteen principal components result


PC1: 84.447%	PC2: 10.149%	PC3: 3.033%	➩ ∑ = 97.63 *%*
PC4: 0.846%	PC5: 0.681%	PC6: 0.249%	➩ ∑ = 1.776 *%*
PC7: 0.188%	PC8: 0.122%	PC9: 0.087%	➩ ∑ = 0.397 *%*
PC10: 0.060%	PC11: 0.055%	PC12: 0.036%	➩ ∑ = 0.151 *%*
PC13: 0.033%			➩ ∑ = 0.033 *%*

These are the total variances explained by the percentage for each principal component of the UTPM-normalized data. Total of 13 principal components (PC1, PC2, PC3, PC4, … PC13) is produced as an outcome of the PCA for our each data-column representation. However, reduced data dimension is the way of selecting the highest variances of principal components among them, higher the value, which determines the most likely principal component, needs to be taken. In this instance, first three principal components (total of 97.63%) are good enough to represent the entire data selected due to the highest variances among others (total of 2.357% = 1.776%+ 0.397%+ 0.151%+ 0.033%).

#### Using PCA to represent the data in a reduced dimensionality form

These first three principal components accounted for 97.629% of the total variances (Table 2). The other principal components (from PC4 through PC13) were sufficiently low that they could be discarded. Thus the first three principal components can be treated as the new, reduced dimension, form of the ILD data.

**Figure 4:**
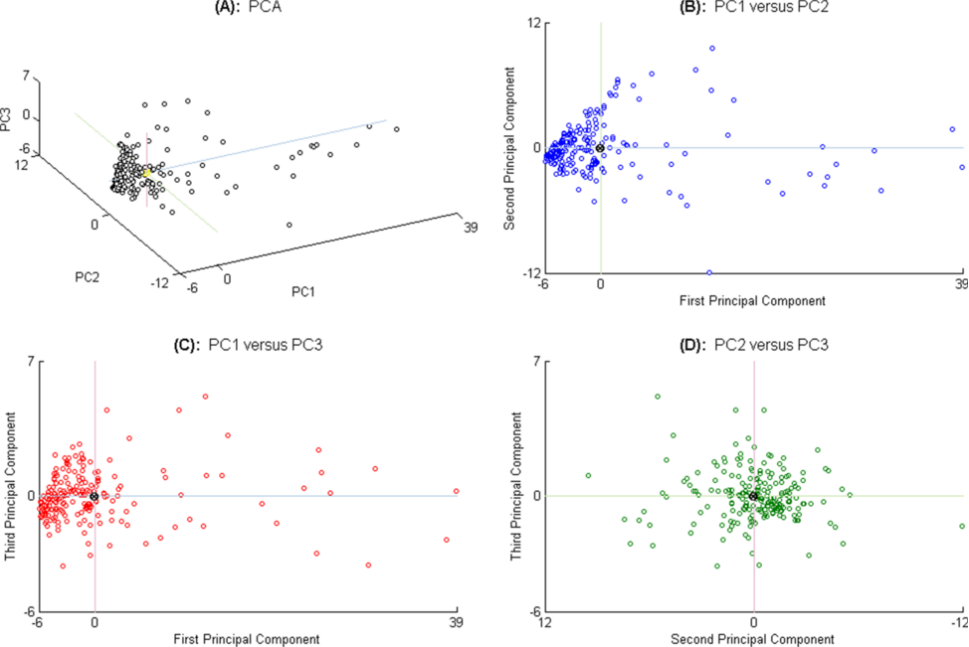
**First three principal components are depicted in 3D.** The selection of first three principal components is decided by the Scree-plot (Figure 3G), and expressed for 208 normalized data (circles) in three-dimensional plot, **(A)**. These transformed values are viewed by pairs in two-dimensional: First and second principal components **(B)**, first and third principal components **(C)**, and second and third components in **(D)**. All zero origins are marked “⊗” for a reference point with the line axes.

The effects of pairwise combination of the first three principal components in two-dimensional projections, namely PC1 vs. PC2, PC1 vs. PC3 and PC2 vs. PC3, are shown in Figures 4B-D. In our three two-dimensional principal component projections, the coefficients are spread widely, (Figures 4B-D), being:

(i) −6 ≤ PC1 (first principal component) ≤ +39

(ii)  −12 ≤ PC2 (second principal component) ≤ +12

(iii)  −6 ≤ PC3 (third principal component) ≤ +7

The values of the first three principal components were projected onto the three axes of a three-dimensional plot in Figure 4A. However, despite accounting for a large part of the variance in the ILD data, the reduced dimension data representation with three principal components was still not sufficient to distinguish the number of classified data. To address this issue, we turned to the third step of our process, Cluster Analysis, to classify the data.

### Cluster analysis result

For Cluster Analysis, the application of *Cophenetic Correlation Coefficient* (CCC) brought us two suitable algorithms (*Cosine* pairwise-distance and *Ward* linkage) as mentioned in Table 3. The suitability of the Ward linkage algorithm for our data is supported by the idea that clusters of multivariate observations are expected to be in an approximately elliptical form [41] and Figure 4 shows that our data distribution is indeed distributed in an elliptical form.

**Table 3:** The assessment of the cluster dissimilarities

**Cophenetic correlation**		**Linkage algorithms**			
**Coefficients (CCC)**		***Single***	***Average***	***Complete***	***Ward***
**Pairwise - distance algorithms**					
***Euclidian***		0.69391	0.77691	0.6625	0.57017
***Seuclidian***		0.75833	0.80679	0.64322	0.48974
***Minkowski***		0.69391	0.77691	0.6625	0.57017
***Mahalanobis***		0.75833	0.80679	0.64322	0.48974
***Cityblock***		0.72678	0.79032	0.57675	0.54419
***Cosine***		0.34928	0.81656	0.73456	**0.83168**

CCC measures the cluster dissimilarities. The most suitable algorithms produce the coefficient which is closer to one “1”. In this case, *Ward* linkage and *Cosine* pairwise-distance algorithms generate a coefficient (0.83168) that is the closest to one ‘1’ among other coefficients.

**Figure 5:**
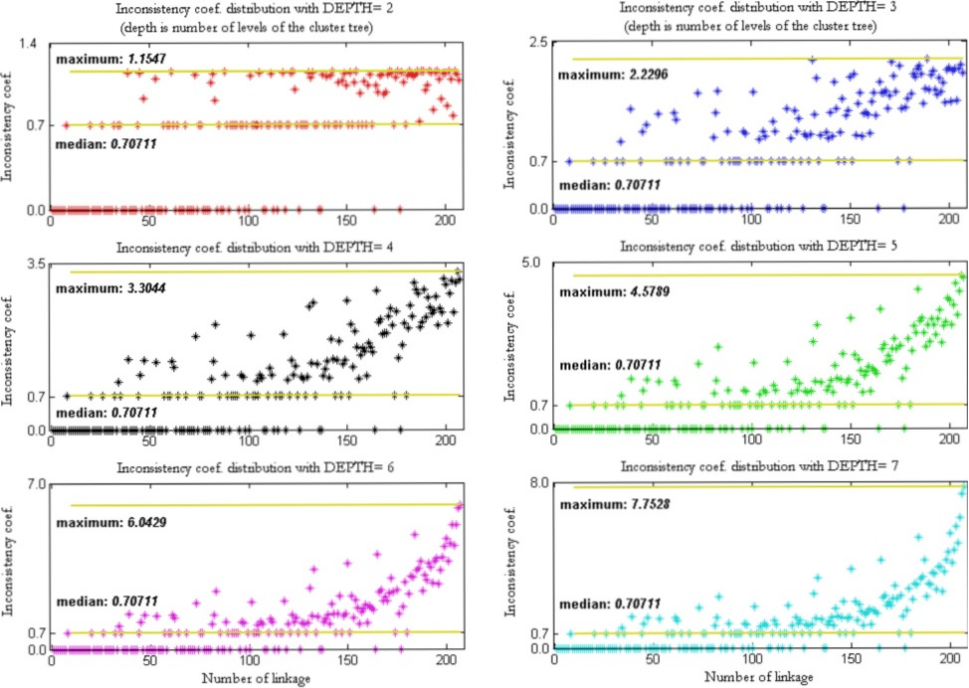
**The selection of inconsistency coefficient.** The Inconsistency Coefficient varies by the number of clusters (as a depth value in Dendrogram) for number of linkage distributions. This distribution becomes in more compact form around the maximum value of Inconsistency Coefficient of seven. This value suggests the cut-off point for the Dendrogram or the number of cluster selection.

Using these two algorithms for the Cluster Analysis led us to investigate the *Inconsistency Coefficient*, Figure 5. From the Inconsistency Coefficient, we found out the natural segregation between the clusters is realized by a certain depth value.

**Figure 6:**
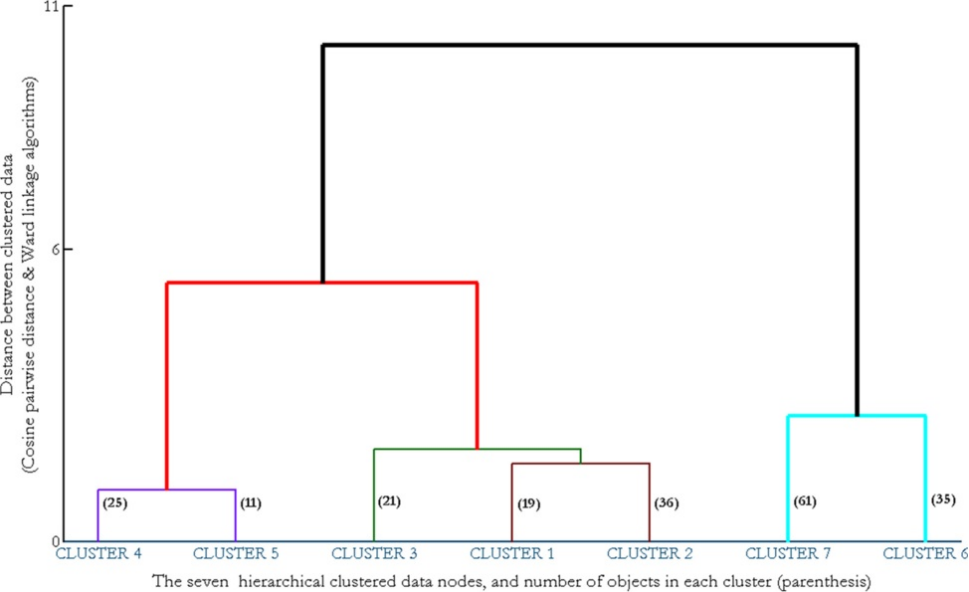
**Seven clustered data represented by the Dendrogram.** This Dendrogram is extracted from Figure 12 with the cut-off point of seven. The homogenous distribution of seven clustered data emphasized by their number of objects (bold parenthesized) for each cluster, i.e. Cluster-4 contains 25 similar ILD functions, which are also close relative of Cluster-6, which consists of 11 similar ILD functions.

**Figure 7:**
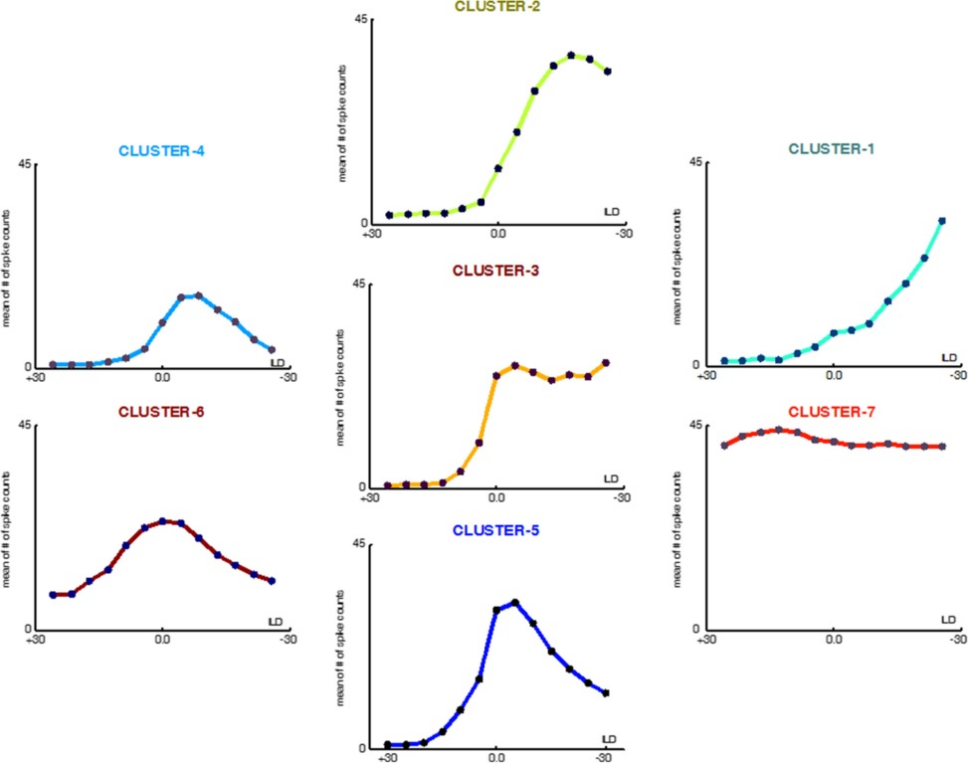
**Seven types of ILD functions observed.** Typical four ideal ILD functions (Figure 8) can easily be perceived among these seven type of ILD functions here; what makes the another three “transitional” cluster findings is significantly important in this study. Type of ILD functions are derived from each clustered data by averaging their objects. All maximum numbers of mean spike counts is scaled up to 45 for a comparison reason. For example, The Cluster-4 shows peak type ILD functions by averaging its (25) objects where the Cluster-6 also shows arisen-peak ILD functions by averaging its (11) objects. These numbers of objects are also shown in Figure 9.

**Figure 8:**
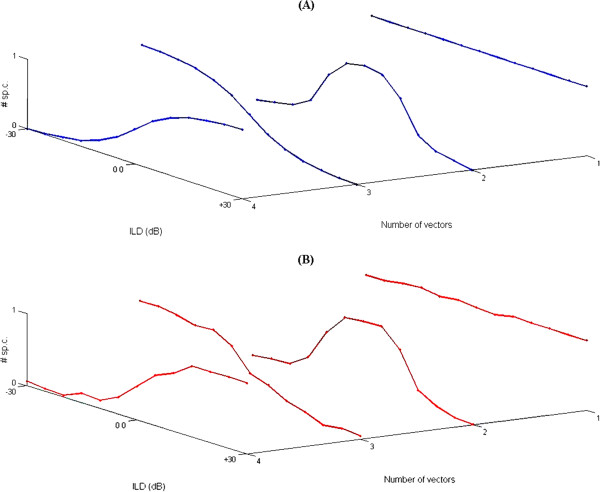
**The four ILD functions.** Typical four ideal ILD functions **(A)**, namely they are, *Sigmoidal* (EI), *Sigmoidal* (IE), *Peak*, and *Insensitive*. These four ILD type representations are slightly perturbed to give more realistic aspect **(B)**. Four ILD patterns are described in numbers of spike counts “#sp.c.” (spikes/ stimulus) which varied between maxima of *‘m’* units (m ∈ ℵ) and minima of *‘0’* zero unit, within -30dB to +30dB interaural level differences.

Cluster Analysis yielded seven clusters of data, each containing a number of objects as shown in the *dendrogram* in Figure 6. We then averaged the objects in each cluster so as to represent the common data characteristics of each cluster with a mean ILD function for that cluster. Figure 7 shows the generic form of the ILD function found in each of these seven clusters; the type of ILD function in each cluster was derived by averaging the ILD functions (the “objects”) making up each cluster. The four-prototypical ILD functions generally reported in the literature (see Figure 8) can easily be perceived among the seven types of ILD functions shown here. The three “new” ILD function types found here are “transition” ILD function and represent the novel finding of significance in this study.

**Figure 9:**
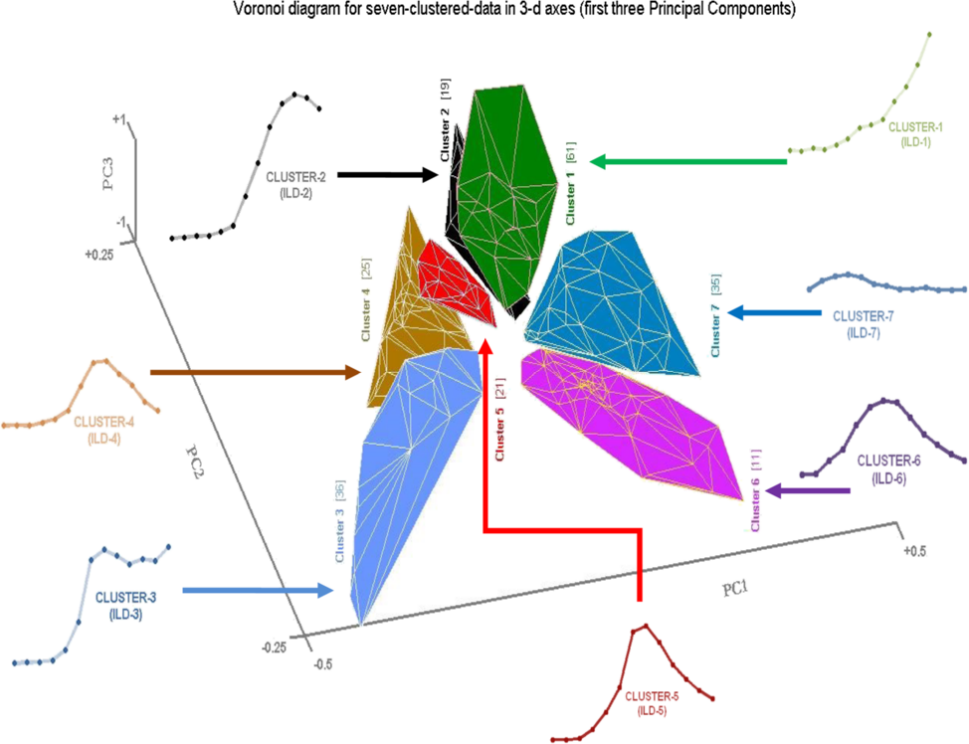
**The Voronoi diagram for seven clustered data.** Each cluster (from Cluster-1 to Cluster-7) holds number of objects (bracketed, ‘n’) and Seven ILD functions ensemble by the averaged for each clustered-data, these are; ILD-1= mean(Cluster-1), ILD-2= mean(Cluster-2) … ILD-7= mean(Cluster-7), from Figure 7. These seven ILD functions are positioned around the Voronoi diagram of clustered data to show the relationship between clustered data and its representation of the ILD function. Seven clustered-data and contained ‘n’ number of objects (‘n’/208) are viewed; Cluster-1 (61/208), Cluster-2 (19/208), Cluster-3 (36/208), Cluster-4 (25/208), Cluster-5 (21/208), Cluster-6 (11/208), Cluster-7 (35/208).

These seven ILD data clusters are also shown in the PCA transformed-data arrayed in three-dimensional space. We applied *Voronoi* analysis (a way of presenting clustered data points by connecting them) to the data arrayed in 3-d PCA space (i.e., using only the first three principal components accounting for maximum variances) to generate clusters separated by clear borders, as shown in Figure 9. The location of these seven clustered Voronoi diagrams in the representation of our data by PCA linear transformation show a very satisfying outcome: that the ILD functions recorded electrophysiologically can be arrayed in a continuum from sigmoidal ILD functions to peak ILD functions to, finally, insensitive (flat) ILD functions. This can be seen in the organization of Figure 9 from Cluster-1 to Cluster-7 in a clock-wise rotation format. As evident, the average value of Cluster-3 data is the sigmoidal ILD function, Cluster-5 data is the peak ILD function and Cluster-7 data is the insensitive (flat) type of ILD function. The averages of the other clustered data functions show nice transitions between those ILD functions for Cluster-3, Cluster-5 and Cluster-7.

## Discussion

### Data normalization conclusion and discussion

We developed prototypical ILD functions to test several normalization techniques. This type of test bench was used for the first time in this field to investigate appropriate normalization techniques. This testing showed that the best method for normalization of ILD functions was the UTPM normalization method.

Generally, in data pre-processing, the normalization procedure is selected to be specific for the application under study, even if it is necessary to improvise by slight adaptation of existing normalization techniques (e.g., multiplication of the mean value of data for our UTPM type of normalization technique for each ILD function). It is true that we did not test all possible normalization methods, e.g., other data normalization methods which are variants of existing ones, such as dividing by a sum of all signals or by the standard error signals after correcting mean values [19]. We also recognize that slight variations of some of the methods used here could give a normalization procedure that would result in a non-linear feature for the data [17]. Finally, in statistics it is a common practice to devise new normalization technique, as has been used to design new normalization technique for microarray data analysis [13], or sometimes different datasets are applied to find the normalization technique that most reduces variations by a comparison with the original data set [12].

We also note that in addition to visually comparing the raw data against the result of applying a selected normalization technique to the data, other methods are also available. Selection of the correct normalization technique can be quantified by examining the quality of the normalization technique and this can be estimated by; (a) calculating the sum of squares of differences between the model and normalization histogram, (b) using Pearson correlation coefficients between the values before and after normalization of data [42]. Such a quantification method for normalization selection is worth investigation, but is beyond the scope of this study.

Despite these constraints, we believe that we have identified an appropriate normalization technique that can be successfully applied to electrophysiologically-recorded neuronal sensitivity functions for ILD, the major binaural cue for azimuthal location of high-frequency sounds.

### PCA discussion

There are several data dimension reduction techniques but to our knowledge, none of them have been applied to the study of ILD sensitivity functions. It is therefore not possible to evaluate the other types of data reduction techniques against the PCA we applied for data reduction of our ILD functions. Instead of comparing other data reduction techniques to PCA usage for the ILD data, we will therefore briefly explain the other types of data reductions techniques and their suitability for use for analysis of ILD sensitivity data.

The PCA is a good linear data analysis tool despite some limitations for data classification studies. There have been some strategies applied to overcome the shortcomings of PCA by implementing higher-order statistics, such as in nonlinear PCA. These include techniques such as *Independent Component Analysis*[43,44], an extension of PCA; *Vector Quantization PCA*[45] or a *Kernel PCA*, a non-linear generalization of PCA cited in [46]. There are even a few new dimensionality reduction procedures such as *Linear Discriminate Analysis*[47], or a combination of PCA and Discriminate Analysis as an efficient dimension reduction technique [34] or more specific applications such as in a PCA mixture model discussed in [48].

In summary, although PCA is limited for use for nonlinear data applications, it is actually helpful to discriminate the linear variations of data from the nonlinear ones.

### Cluster analysis discussion

Cluster Analysis is a broad area in the field of data classification and many clustering algorithms have been developed for many classification applications in diverse scientific areas. These algorithms have some advantages and disadvantages. The following paragraphs will discuss a few Cluster Analysis algorithms and the data to which these have been applied for categorization. This scheme will not tell us which algorithm would be better than the other but may help divulge which algorithm is more applicable to a specific problem since clustering algorithms are application orientated statistical tools.

#### What is important in cluster analysis?

Cluster Analysis is achieved by a specific algorithm designed for a specific application [23]. There have been over 100 clustering algorithms available for Cluster Analysis [49]. Unfortunately, there is no generic Cluster Analysis algorithm that can be used to give the best solution for all types of data [50]. It is also not practical to design a clustering algorithm for each new application. Thus it is best to choose an algorithm that has been used for a similar type of application and is also a less time-consuming approach, and is a process that is widely accepted in the field of study of Cluster Analysis. In the end, three things define the importance of Cluster Analysis: (a) Speed, (b) reliability and (c) consistency [49].

## Conclusions

In this study we found that the UTPM normalization method was the best data normalization method applicable for ILD sensitivity functions. PCA was used to reduce the dimension of the large number of multivariable data that made up the 208 ILD functions we recorded from the midbrain auditory nucleus, the ICc, of the rat. The transformations used variances of highly correlated variables, and it was found that the first three principal components (i.e., variances) were good enough to represent our normalized data. The variances are explained in terms of percentage accounted for, as well as indicated in the Scree-plot and both showed that more than 84% of the transformed data are accounted for by the first three principal components (Total variance explained > 97%). In the process our transformed data were converted from a 13x208 matrix into a form of 3x208 matrix.

Hierarchical Cluster Analysis with the agglomerative technique was used to determine the number of clusters of homogenous data objects in our data. For this analysis, we combined visual and automated Cluster Analysis of the full lot of ILD functions we investigated. We then applied common *dendrogram* and data cluster techniques that were available at MATLAB version 6.5, Statistic Toolbox version 4.1.

Several pairwise-distance and linkage algorithms were applied to our 3x208 transformed data. The best combination of these was determined as the one generating the highest CCC and this was the Cosine pairwise-distance algorithm combined with the *Ward* linkage algorithm. This pair of algorithms was used to plot dendrograms to visually present the distribution of the clusters of homogenous data.

To determine the cut-off for the number of clusters the Inconsistency Coefficient from the linkage algorithm application was applied to determine a depth value of three, and resulting in a maximum of seven cluster types. Averages were determined from all ILD functions in each of the seven clustered data (from visual and automated analysis) to identify the prototypical ILD function in that cluster. Then, statistical data analysis methods were used to differentiate between the ILD functions. The result showed seven different prototypical ILD functions, obtained from the three broad categories of ILD functions, namely peak, sigmoidal (two types “EI” and “IE” of them) and insensitive ILD functions, as in Figure 8. More than 80% of the electrophysiological data were of the peak and sigmoidal type of ILD functions. These analyses were completely congruent with the Cluster Analysis and the seven ILD function types from statistical analyses corresponded very well with the seven ILD function types determined by Cluster Analysis.

In addition:

(i) Cluster Analysis was used to determine the number of data groups after PCA.

(ii) Cluster Analysis is a way of segregating data into meaningful subgroups or clusters.

(iii) Clustered data can be obtained in two ways, supervised and unsupervised data clustering.

(iv) There are several clustering algorithms assist for several different types of data clustering methods (K-Means, Hierarchical, Latent Class Analysis, Latent Profile Analysis, and Factor Mixture Model). Hierarchical and Agglomerative types of Cluster Analysis are the most common techniques that applied, where the number of sub-groups and their contents (number of data to be formed) are unknown.

(v) The hierarchical agglomerative technique is commonly used and is the most suited to our data. This method involves four steps [51]:

a) Sequentially merge the most similar cases in the N×N similarity matrix, where "N" is the number of objects.

b) This visual representation of the sequence of merged clusters can be illustrated in a tree type structure called a "dendrogram".

c) “N-1” steps are required as numbered of clustered nodes.

d) All cases are merged into one group.

(vi) Once the number of clusters and their number of objects are defined, then the result can either be illustrated or tabulated to finalize the data classification solution.

## Methods

### Data normalization method

#### Generating prototypical ILD functions

Our own database and an extensive literature review showed that there are four prototypical ILD functions in [different] neurons at all levels of the brain beyond the brainstem [52-55]. These four prototypical functions (Figure 8) consist of two Sigmoid response functions where neuronal responses vary in a sigmoid function with variations in ILDs but with the plateau of responses in one ILD range (favouring one ear) or the other (favouring the other ear), a Peaked response function where neuronal responses are peaked at some ILD that would arise from frontal space, and Insensitive response function where neuronal responses vary little with ILDs. Each of these four broad response categories encompasses functions that can vary in metrics defining the features of that ILD function type, e.g. position of peak or the slope along ILD axis, the steepness of the slope along ILD axis – all of which are features that have been variously discussed to be defining information-bearing elements for deriving azimuthal location of a sound source [56].

In the simulated ILD sensitivity functions, neuronal responses were represented on a scale from ‘0’ to ‘100’ (“*m*”, maximum response count). This normalized scale allowed us to simulate ILD functions in absolute values. These minimum ‘0’ and maximum ‘100’ values (spikes/stimulus) are also selected for all normalization test bench to give a good comparison in the result.

**Figure 10:**
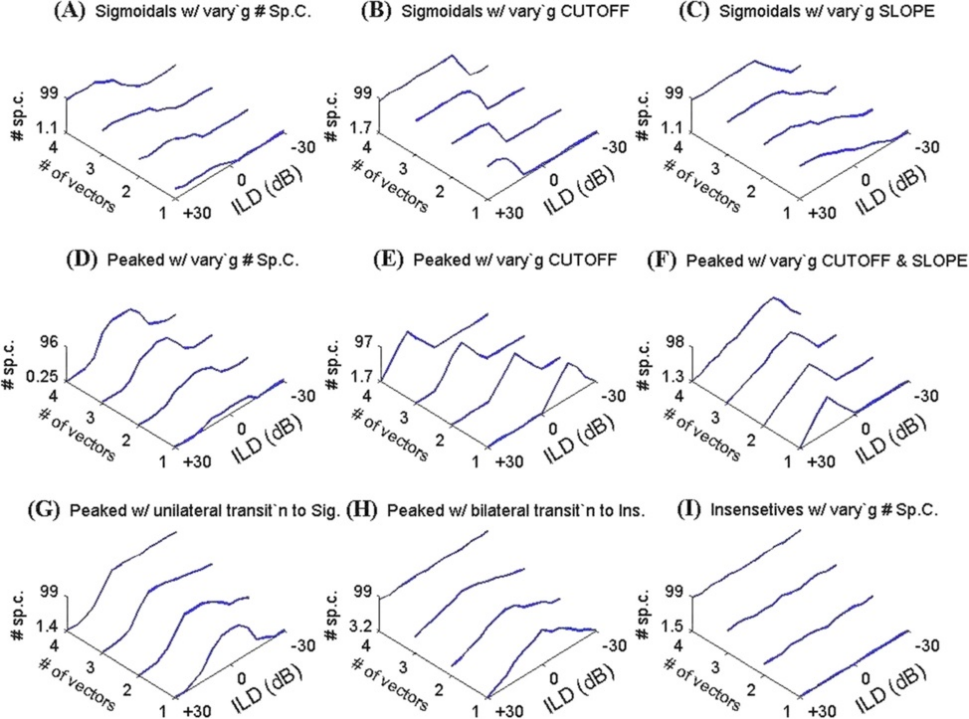
**The simulation of nine possible ILD functions.** Nine possible ILD functions are generated from four typical ILD sensitive function variations (Figure 8B) These are; *Sigmoidals* with varying number of spike count (# sp.c.) spikes/ stimulus **(A)**, position of the cut-off **(B)**, the steepness of the slope **(C)**, and four *Peaked* with varying number of spikes/stimulus **(D)**, the cut-off **(E)**, the cut-off & slope **(F)**, and *Peaked* with unilateral transition to *Sigmoidal***(G)**, and *Peaked* with bilateral transition to *Insensitive***(H)**, and four *Insensitive* with varying the number of spike count spikes/ stimulus **(I)**.

To produce more realistic looking ILD functions we applied a small and statistically-insignificant perturbation to deform the shape of the nine ILD functions, [33]. All points in the data groups (*viz*., numbers of spike counts) were arbitrarily perturbed within ± 6% of the original values. The ± 6% perturbation range was determined from initial visual inspection of different test ranges which showed that perturbation by < 6% made the ILD functions still look too ideal and perturbation by > 6% made it too easy to confuse different ILD patterns. For example, a 7% increase in one part (and 7% decrease in another part) of the insensitive ILD function (v#1s of Figure 10A and I) made it look like a Sigmoidal ILD function.

Data perturbation was carried out in three steps:

(i) First generate 13 random numbers varying between −0.06 and +0.06 for every unit (i.e. ±6 for the maximum of 100 spike counts, viz., 6% variation),

(ii) then add the 13 random numbers arbitrarily to each ILD pattern (each ILD pattern contains 13 numbers and each number presents the number of spike counts), and

(iii) finally, monitor the previous steps to verify that perturbed data validly fit in the range from minimum to maximum (e.g., log normalization may produce errors for some values if divided by zero).The ±6% perturbation was applied to the nine ILD functions to produce the final test bench.

### Data dimension reduction method: PCA

The PCA algorithm is based on three-step procedure. The objective of this algorithm is to find the principal components with the highest variances, Equation 1.

*STEP 1*: Finding a covariance matrix from the ILD patterns of an input matrix,

*STEP 2*: Using covariance matrix to find eigenvectors, and

*STEP 3*: Using eigenvectors to find principal components.

This three-step procedure is formalized in Equation 1, [22,46,57-59].

(1) $$\begin{array}{l} A=\frac{1}{N}\sum_{k=1}^{N}{\left(x_{k}-\mu \right)}^{T}\cdot \left(x_{k}-\mu \right)\\ A\cdot \upsilon =\lambda \cdot \upsilon \Rightarrow \left(A-\lambda \cdot I\right)\cdot \upsilon =0\\ y=\upsilon^{T}\cdot x \end{array}$$

The PCA algorithm, where vector mean: “μ*”*, number of sample: “N”, largest Eigenvalues (covariance matrix) of “*A*”: “λ”, eigenvectors “υ”, principal components: “*y*” and set of centered input vectors: “x”, and the unit matrix: “I”.

The most common usage of PCA is utilizing similar type of data groups that can be observed in a two or more dimensional space. These dimensional space axes are named as principal components. The number of principal components can be expressed as a percentage of the highest variances in Equation 2.

(2) $$\mathrm{principal}\;\mathrm{components}\;\left(\%\right)=\upsilon \frac{100}{\sum \upsilon }$$

where, total variability can be explained by each principal component in percentage, with the highest variances “υ”.

In practice, ILD sensitivity functions are statistically multivariate data, which can be exhibited as a single matrix. The dimension of this matrix form can be reduced with the aid of PCA. The PCA actually reconstructs the data on an orthogonal basis such that the columns represent the principal components of the projected values. The correlation between the first column and the other column represents the variance. The values of variances are greater between the first column and other columns and the variance values are reduced between the second column and other columns and then further reduced between the third column and other column, and so on. It is therefore an elegant way to represent the multivariable data with only few variables, which corresponds to just the first few columns.

### Cluster analysis method

**Figure 11:**
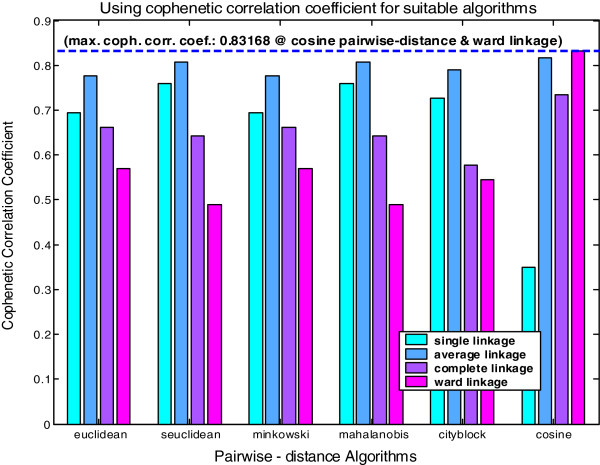
**Cophenetic Correlation Coefficient (CCC) utilization for dendrogram.** The CCC determines suitable algorithms for dendrogram the combination of 24 different (six pairwise-distance and four linkage) algorithms used to find most two suitable algorithms for the dendrogram plot. The optimization criteria using CCCs applied for 24 combined algorithms are also shown in Table 3. The algorithms used for pairwise-distance and linkage methods and CCC application operated from MATLAB version 6.5.

Figure 11 shows the CCC for the Cluster Analysis. The CCCs show that there are similar changes in the six linkage algorithms and four pair-group pairwise-distance algorithms (see light-blue, dark-blue, purple and pink colored bars). As a measure of the distortion between clusters, using CCC offers suitable algorithms for both linkage and pairwise-distance algorithms. The CCCs are tabulated in Table 3 where the maximum value indicates the best selection of the pairwise-distance and linkage algorithms combinations for the dendrogram (below).

#### Dendrogram

The representation of clustering derived from the Cluster Analysis can be visualized in a tree-shaped graphical representation termed a *dendrogram*[51]. The vertical axes represent the clustered data groups in pairwise-distances, and horizontal axes represent the predefined number of clusters.

**Figure 12:**
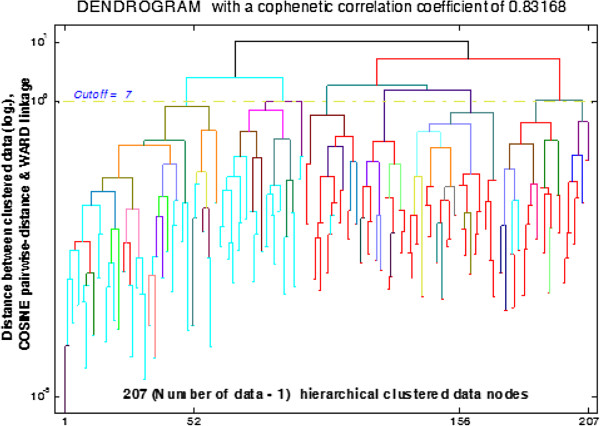
**Dendrogram for cluster composition.** Using CCC as a dissimilarity cluster measurement to select *Cosine* pairwise-distance and *Ward* linkage algorithms for cluster composition in the Dendrogram. The horizontal axis represents the all observation of subjects with 207 (208–1) numbers of clustered nodes; vertical axis represents the distance between the observed subjects in a logarithmic scale from 10^-5^ to 10^1^.

The cluster organization is depicted in the dendrogram in Figure 12 where Cosine pairwise-distance and Ward linkage algorithms are used (other algorithms were also tested as the worst case analysis but, because the data-nodes distribution were not homogenously spaced, these results are not presented). It shows all 207 possible cluster connections (calculated from the total number of data, 208 minus one). In this figure, all clusters are shown in a linear form and the distances between the observations in a logarithmic scale. Clearly the number of clusters and the selection of threshold depend on the line as a cut-off point. From the visualization the cut point of seven clusters was arbitrarily selected. However, it should be noted that the selection of a cut point between clusters can be automated by using the Inconsistency Coefficient (Figure 5).

The application of the Inconsistency Coefficient with different number of levels (depth) of the cluster tree is shown in Figure 5. This helps to comprehend the cluster tree distribution in the dendrogram, (Figure 12). The denser the distribution’ more likely that less similar objects are linked to each other; for example; the depth of seven (i.e. seven levels of cluster tree) explains how dissimilar objects are linked to each other, on the other hand three levels of cluster tree in shows the objects are began spreading sparsely around the median value. Note that the median values of Inconsistency Coefficients are always the same due to the fact that the same clustering algorithms (Cosine and Ward) were used for the linkage and pairwise-distance distribution and then applied here to these different numbers of clustering trees.

## Materials and method: source of data

In this study, 208 extracellular ILD sensitivity functions were recorded from the left of ICc (Central nucleus of the Inferior Colliculus) cells of male rats. Data collection was carried out in a series of experiments conducted by the second author prior to this present study. These data have not been published and formed the data set used for the present modelling study. Only a brief description is given of the procedures used for that data collection.

### Animal preparation and surgery

#### Ethics statement

All animal experiments were approved by the Monash University Department of Animal Ethics Committee (PHYS/1997/03 and PHYS2000/22) and conducted in accordance with protocols designed by the National Health and Medical Research Council of Australia for the ethical care and welfare of animals under experimentation.

#### Animal preparation

Adult male Sprague–Dawley and Long-Evans SPF (Specific Pathogen Free) male rats (weight range, 250 to 450 grams) were obtained from the Monash University Animal Services for all experiments. Each rat was initially anesthetized with an intraperitoneal injection of 60mg/ml pentobarbital sodium (Nembutal; 0.1 millilitres/ 100 grams of body weight). Then Xylaze-Saline solution (0.1 millilitres of 1:1) was administered intramuscularly as a muscle relaxant and as an analgesic. Thereafter, throughout the experiment, hourly doses of 0.1 millilitres Nembutal and 0.05 millilitres Xylaze-Saline solutions were injected sub-cutaneously or intra-peritoneally to keeping the rat in deep anesthesia. Body temperature was maintained at 37.5±0.5°C by a rectal probe feedback-controlled electrically heated blanket.

Once deep anaesthesia was established (as evidenced by the absence of withdrawal reflexes to strong noxious pinching of the forepaw as well as absent palpebral reflexes), the rat was transferred to a sound proof room and tracheotomized (a cannula surgically inserted into the trachea) to facilitate mechanical ventilation. Artificial ventilation with room air was adjusted according to the body weight of the rat, with a respiratory rate of 80 ~ 90 breaths/minute and a tidal volume of 3 ~ 4.5 millilitres.

Throughout the experiment, anaesthesia status was monitored through continuous recording of the electrocardiogram (ECG), and electromyogram (EMG) activity from forearm muscles on an oscilloscope as well as through a speaker. Depth of anesthesia was also checked at regular hourly intervals by checking for the presence of withdrawal reflexes to noxious stimuli by pinching of the forepaw and the presence of pupillary dilatation.

#### Electrophysiological recording

Under deep anesthesia a midline incision was made in the skin from top of the rat's skull then cleared of any connective tissues to expose the skull. The pinnae were removed and the ear canals were transected close to the tympanic membrane. A small hole was drilled in the skull at a point over the frontal lobes to allow a metal bar to be affixed by a screw through the bar and into the skull hole. The screw-metal bar system was fortified by a dental acrylic. The position of stabilising metal bar could easily be oriented to give the rat’s head any desired angle. A second hole, approximately 3 mm in diameter, was then drilled over the left occipital lobe of the rat’s skull to allow for an insertion of the recording electrode which would be advanced through the overlying cortex to the ICc. Silicone-oil was applied to the exposed surface of the cortex to prevent it drying out.

Parylene coated tungsten tip microelectrodes (A-M Systems, Inc., WA, USA) with impedance of 2 MΩ were mounted on a micromanipulator mounted on a remotely-controlled steeping-motor drive assembly on a series of translators and goniometers, and the micromanipulator was controlled electronically from outside the sound-proof room. The remotely controlled microelectrode was placed to contact the left cortical surface and then advanced through the cortex to the left IC. Microelectrode penetrations were made into the cortex around positions ~1.1 mm anterior and ~1.7 mm lateral of lambda.

### Data collection

#### Action potentials recording

The remotely-controlled microelectrode was slowly advanced from outside the sound proof room in 5~10 micrometer steps through the cortex to locate a well-isolated cell in the ICc. Identification of the recording locus in the ICc was facilitated (a) by the observation of the expected tonotopic organization as the electrode was advanced through the putative ICc, and (b) the pattern of short latency robust responses to tone stimuli at different frequencies and intensities both binaurally and monaurally.

Action Potentials (APs) were recorded only from well-isolated single cells in ICc, with a signal-to-noise ratio of at least 4:1 between the well-isolated APs and other activity. The output of the microelectrode was first passed amplifiers (preamplifier with the gain of 10, and amplifier with the gain of 100) to the band-pass filter (cut-off frequencies from 100 Hz and 10 kHz) then through the graphic equaliser for shaping the pulse of the APs, and the APs were also observed by an oscilloscope. These APs were digitized by a Schmitt trigger based level detection circuit for a real-time recording. The real-time data with time-stamp information were both saved to the files on to a Personal Computer. In all recordings, the AP waveform was monitored continuously online to ensure recording fidelity and that there was no contamination by activity from other cells.

#### Acoustic stimuli and determination of the characteristic frequency of a cell

Acoustic stimuli were generated by a computer controlled two channel digital synthesiser systems (TDT System II), which were cascaded with digital attenuators. Outputs from the digital attenuators were separately routed to two input channels of HD535 Sennheiser speaker in homemade couplers. The speakers were connected to two sound-delivery tubes, which were placed in the rat’s external auditory meatus of both ears.

Once a cell was sufficiently well isolated, the characteristic frequency (CF; frequency of greatest sensitivity) and the threshold at CF were identified from audio-visual criteria with manual control of the tonal stimuli. This was confirmed by recording responses across a very wide frequency-intensity matrix using gated tone bursts, shaped with a 4 ms (milliseconds) rise-fall time, with variable duration between 50–200 ms depending on the test cells response profile. Cells with only onset components were tested with 50 ms tone bursts, cells with sustained components were tested with 100 or 150 ms tone bursts, and cells with late components were tested with 200 ms tone bursts. (Onset component were classed as responses occurring in the first 50 ms of tone burst, Sustained components were responses from 100 ~ 150 ms, and Late components were responses from 200 ms.)

#### Determining ILD sensitivity functions

Electrophysiological recordings of ILD sensitivity were obtained from a total of 208 cells from the ICc, (see Additional file 1). The stimuli were always CF stimuli gated as described above, with variable duration between 50–200 ms depending on the test cells response profile. The duration of each tone was equal to 50 ms for cells with only onset components, 100 or 150 ms for cells with sustained components, or 200 ms for cells with late components.

ILD sensitivity was tested using the Average Binaural Intensity-constant method [60-62]. In this method the average binaural level is maintained constant at some base level and the sound levels in the two ears are systematically varied around this base level to mimic the origin of a sound source from different positions around the head [63]. In this study ILDs varied from being 30 dB louder in one ear (i.e., Ear 1 = ABI +15 dB, Ear 2 = ABI-15 dB), through 0 dB ILD (both ears = ABI) through to being 30 dB louder in the other ear (i.e., Ear 1 = ABI-15 dB, Ear 2 = ABI+15 dB), in 5 dB ILD steps. These ILDs are designated as ranging from +30dB SPL to -30dB with 5dB intervals, and are calculated by the difference between contralateral and ipsilateral levels. Thus, positive ILDs indicate that the sound was louder in the contralateral ear and negative ILDs indicate that the sound was louder in the ipsilateral ear. In each block, the stimuli were alternated so that a larger contra-lateral intensity was followed by a larger ipsilateral intensity to prevent the cell from fatiguing.

The ABI constant method has been used in similar studies in different brain regions [60-62]. An alternative to ABI constant method, excitatory monaural intensity (EMI) – constant method, has been discussed in the context of recordings from primary auditory cortex, and may have some advantages for interpreting peaked ILD function data but not for non-monotonic functions [64].

## Abbreviations

ABI: Average binaural intensity; APs: Action potentials; CCC: Cophenetic correlation coefficient; CF: Characteristic frequency; EI: Excitatory to the ipsilateral ear and inhibitory to the contralateral ear; IE: Inhibitory to the ipsilateral ear and excitatory to the contralateral ear; ILDs: Interaural level differences; PC: Principal component; PCA: Principal component analysis; UTPM: Unit total probability mass.

## Competing interests

Both authors have no competing interests to declare.

## Authors’ contributions

BU performed all data analyses and was primarily responsible for writing the paper. RR performed the experiments for neural data collection from the auditory midbrain, and provided some assistance in writing the paper. Both authors read and approved the final manuscript.

## Supplementary Material

Additional file 1 **The raw data.** The matrix formation of an unprocessed data is based on the Electrophysiological recordings of ILD sensitivity were obtained from a total of 208 cells from the ICc. Click here for file
