# Supplementary material for: The discrimination of interaural level difference sensitivity functions: development of a taxonomic data template for modelling
Source: BMC Neurosci. 2013 Oct 7;14:114. doi: 10.1186/1471-2202-14-114 (PMC4126173; doi:10.1186/1471-2202-14-114)
Supplement: Additional file 1 — The raw data. The matrix formation of an unprocessed data is based on the Electrophysiological recordings of ILD sensitivity were obtained from a total of 208 cells from the ICc. [file 1471-2202-14-114-S1.pdf]

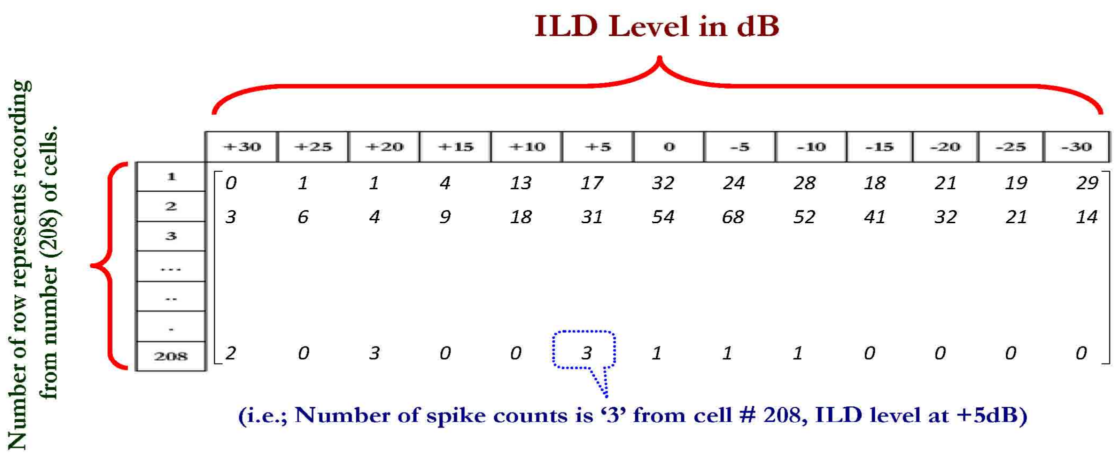

CELL NAME-NUMBER		I L D (Interaural Level Differences) Level in deciBell												
		(+30)	(+25)	(+20)	(+15)	(+10)	(+5)	(0)	(-5)	(-10)	(-15)	(-20)	(-25)	(-30)
'B02U2N60'	1	0.00	1.00	1.00	4.00	13.00	17.00	32.00	24.00	28.00	18.00	21.00	19.00	29.00
'B03U2N35'	2	3.00	6.00	4.00	9.00	18.00	31.00	54.00	68.00	52.00	41.00	32.00	21.00	14.00
'B03U2N40'	3	14.00	12.00	17.00	22.00	26.00	31.00	29.00	32.00	26.00	30.00	31.00	32.00	35.00
'B03U2N55'	4	28.00	33.00	38.00	38.00	39.00	36.00	31.70	35.00	32.00	31.00	32.00	38.00	40.00
'B03U2N70'	5	27.00	27.00	31.00	29.00	23.00	27.00	29.30	28.00	35.00	34.00	44.00	45.00	43.00
'B04U1N35'	6	18.00	18.00	18.00	23.00	32.00	33.00	36.70	33.00	33.00	35.00	40.00	43.00	39.00
'B04U1N50'	7	9.00	20.00	28.00	30.00	35.00	30.00	32.70	29.00	31.00	30.00	32.00	32.00	41.00
'B04U1N65'	8	25.00	23.00	22.00	29.00	24.00	25.00	25.70	26.00	21.00	21.00	19.00	20.00	20.00
'B04U1S35'	9	0.00	0.00	0.00	2.00	8.00	14.00	15.30	19.00	30.00	45.00	59.00	75.00	81.00
'B04U1S50'	10	9.00	14.00	10.00	12.00	15.00	14.00	20.30	36.00	56.00	82.00	75.00	89.00	97.00
'B04U1S65'	11	4.00	4.00	11.00	10.00	15.00	18.00	17.00	15.00	10.00	40.00	39.00	34.00	46.00
'B04U2N35'	12	0.00	0.00	0.00	0.00	0.00	0.00	3.00	15.00	19.00	20.00	19.00	13.00	6.00
'B05U1S60'	13	5.00	20.00	18.50	21.50	22.50	15.00	13.80	16.50	30.50	36.00	44.50	38.50	30.50
'B05U2S55'	14	31.00	21.00	15.00	21.00	31.00	25.00	23.30	15.00	14.00	11.00	13.00	11.00	11.00
'B05U2S75'	15	111.00	118.00	98.00	102.00	104.00	86.00	70.70	45.00	34.00	26.00	21.00	24.00	22.00
'B05U3N10'	16	3.00	1.00	3.00	1.00	4.00	4.00	10.00	10.00	10.00	16.00	29.00	28.00	27.00
'B05U3N30'	17	3.00	2.00	1.00	3.00	3.00	1.00	4.70	6.00	9.00	11.00	14.00	6.00	10.00
'B05U3N50'	18	22.00	22.00	18.00	12.00	19.00	29.00	24.70	29.00	16.00	8.00	10.00	6.00	1.00
'B05U3S10'	19	21.00	25.00	32.00	22.00	32.00	25.00	29.70	26.00	83.00	130.00	164.00	167.00	144.00
'B05U3S30'	20	51.00	61.00	83.00	78.00	71.00	45.00	51.00	47.00	39.00	39.00	43.00	45.00	47.00
'B05U3S50'	21	22.00	22.00	18.00	12.00	19.00	29.00	24.70	29.00	16.00	8.00	10.00	6.00	1.00
'B07U2N40'	22	1.00	4.00	15.00	20.00	20.00	22.00	28.00	25.00	25.00	22.00	20.00	21.00	20.00
'B07U2N55'	23	19.67	20.33	20.00	20.33	20.00	21.00	22.00	21.33	21.33	20.67	20.67	20.33	20.33
'B07U2N70'	24	20.00	20.00	19.00	19.00	18.00	18.00	20.00	21.00	20.00	20.00	20.00	20.00	20.00
'B07U2S40'	25	4.00	3.00	6.00	3.00	7.00	11.00	9.00	4.00	9.00	2.00	2.00	1.00	3.00
'B07U2S55'	26	3.67	0.67	1.67	1.67	2.00	3.67	5.00	7.00	5.00	4.00	3.00	1.00	1.00
'B07U2S70'	27	0.00	1.00	1.00	1.00	1.00	5.00	5.00	3.00	4.00	2.00	1.00	0.00	0.00
'B07U4N50'	28	3.00	0.00	2.00	1.00	2.00	1.00	0.70	5.00	3.00	1.00	0.00	1.00	2.00
'B07U4S50'	29	0.00	1.00	0.00	0.00	3.00	12.00	36.30	42.00	35.00	21.00	28.00	30.00	18.00
'B07U5N40'	30	27.67	23.67	20.00	19.67	16.33	16.33	16.67	11.67	13.67	10.67	14.33	8.67	9.00
'B07U5S40'	31	44.67	40.00	36.33	39.67	48.33	62.67	74.57	90.67	83.67	77.33	53.00	41.33	28.67
'B08U1N60'	32	0.00	0.00	0.00	0.00	1.00	2.00	12.30	21.00	18.00	19.00	7.00	3.00	1.00
'B08U2N30'	33	27.00	24.00	23.00	27.00	23.00	27.00	23.30	24.00	25.00	23.00	23.00	19.00	14.00
'B08U2S30'	34	150.00	155.00	159.00	168.00	161.00	153.00	170.30	164.00	165.00	160.00	164.00	164.00	181.00
'B08U3N30'	35	3.00	4.00	1.00	4.00	2.00	5.00	6.30	8.00	10.00	5.00	7.00	6.00	3.00
'B08U3N40'	36	10.60	12.00	5.40	10.40	8.80	9.60	11.46	9.00	8.00	10.00	9.40	11.60	14.20
'B08U3N50'	37	6.67	4.00	3.67	3.67	4.33	3.00	2.90	4.00	3.67	3.33	4.33	1.67	4.00
'B08U3N60'	38	3.00	2.00	3.00	3.00	3.00	5.00	2.30	1.00	2.00	1.00	5.00	6.00	3.00
'B08U3S30'	39	108.00	111.00	116.00	99.00	108.00	124.00	112.70	129.00	115.00	101.00	100.00	109.00	109.00
'B08U3S40'	40	108.40	104.60	104.80	103.60	97.00	92.20	96.98	84.80	80.80	78.40	77.00	85.60	102.00
'B08U3S50'	41	97.00	97.33	92.00	85.67	89.33	87.00	88.10	87.00	85.00	85.67	83.00	80.33	82.33
'B08U3S60'	42	114.00	103.00	107.00	95.00	95.00	103.00	114.70	113.00	100.00	104.00	96.00	95.00	112.00
'B09U1S60'	43	65.00	60.00	54.00	48.00	54.00	66.00	85.70	75.00	94.00	83.00	86.00	81.00	78.00
'B09U2S40'	44	36.00	26.00	25.00	19.00	18.00	12.00	20.00	13.00	16.00	17.00	8.00	13.00	17.00
'B09U3N65'	45	0.00	0.00	0.00	1.00	4.00	9.00	3.70	8.00	6.00	5.00	4.00	1.00	3.00
'B09U3S65'	46	7.00	8.00	6.00	4.00	7.00	14.00	35.00	43.00	41.00	44.00	59.00	70.00	103.00
'B09U4N45'	47	0.00	0.00	0.00	0.00	0.00	1.00	4.70	10.00	11.00	13.00	17.00	17.00	12.00
'B09U4N75'	48	0.00	2.00	2.00	5.00	7.00	9.00	11.00	15.00	18.00	16.00	12.00	7.00	9.00
'B09U4S45'	49	5.00	5.00	1.00	8.00	4.00	0.00	9.00	9.00	15.00	5.00	1.00	0.00	5.00
'B09U4S75'	50	6.00	2.00	3.00	2.00	4.00	14.00	23.30	22.00	30.00	30.00	34.00	35.00	51.00
'B09U5N60'	51	0.00	0.00	0.00	0.00	5.00	9.00	10.70	12.00	10.00	9.00	9.00	3.00	4.00
'B09U6N65'	52	1.00	0.00	3.00	11.00	12.00	8.00	15.70	15.00	13.00	10.00	4.00	3.00	0.00
'B10U1N40'	53	0.00	0.00	0.00	0.00	0.00	5.00	19.00	24.00	23.00	22.00	22.00	24.00	25.00
'B10U1N60'	54	0.00	0.00	0.00	0.00	0.00	17.50	34.85	38.00	40.00	37.00	38.50	39.50	39.00
'B10U1N75'	55	0.00	0.00	0.00	0.00	3.00	24.00	34.00	40.00	38.00	32.00	35.00	31.00	28.00
'B10U2N60'	56	0.00	0.00	0.00	0.00	0.00	0.00	3.30	7.00	2.00	3.00	0.00	1.00	0.00

'B10U2S60'	57	0.00	0.00	2.00	4.00	14.00	19.00	29.30	42.00	36.00	26.00	16.00	17.00	19.00
'B10U3N60'	58	0.00	0.00	0.00	0.00	0.00	0.00	0.00	15.00	33.00	39.00	36.00	45.00	35.00
'B10U3S60'	59	0.00	2.00	3.00	1.00	1.00	0.00	10.70	14.00	28.00	33.00	31.00	47.00	58.00
'B11U1S57'	60	51.40	49.20	56.00	58.40	64.20	63.60	53.60	56.60	56.00	45.60	31.20	31.20	27.20
'B11U4N25'	61	4.00	15.00	20.00	23.00	22.00	27.00	25.70	30.00	36.00	38.00	38.00	35.00	35.00
'B12U1S65'	62	20.50	14.00	12.50	12.50	24.00	47.50	74.50	83.50	93.00	95.00	121.00	120.50	114.50
'B12U2N65'	63	6.00	6.00	12.50	14.00	17.00	18.50	21.85	23.00	24.50	23.00	23.00	21.00	17.50
'B12U2S65'	64	12.00	13.00	25.00	33.50	39.00	44.50	36.85	55.00	81.50	97.00	106.00	111.50	99.50
'B12U3N20'	65	6.00	2.67	3.67	7.33	12.00	20.33	25.87	25.33	25.33	23.33	25.33	22.33	23.00
'B12U3S20'	66	0.00	0.00	3.00	6.00	22.00	36.00	30.70	25.00	15.00	17.00	20.00	26.00	27.00
'C17U1N30'	67	0.00	0.00	1.00	0.00	2.00	15.00	29.00	30.00	34.00	41.00	38.00	44.00	44.00
'C17U1N50'	68	0.00	0.00	0.00	1.00	2.00	2.00	30.00	38.00	42.00	50.00	54.00	52.00	58.00
'C17U1N70'	69	0.00	1.00	1.00	1.00	2.00	1.00	12.00	14.00	31.00	44.00	36.00	35.00	35.00
'C17U2N30'	70	0.00	0.00	0.00	6.00	12.00	23.00	33.00	35.00	34.00	25.00	24.00	22.00	18.00
'C17U2N40'	71	0.00	1.00	3.00	6.00	9.50	18.00	30.00	24.50	20.50	17.50	12.50	7.50	2.50
'C17U2N50'	72	0.00	0.50	0.50	0.50	2.00	5.50	23.00	19.50	17.50	11.50	8.50	5.00	3.00
'C17U3N30'	73	3.00	1.00	0.00	1.00	0.00	5.00	10.00	11.00	9.00	8.00	4.00	2.00	1.00
'C17U3N40'	74	1.00	0.00	0.00	0.00	0.00	3.00	15.00	20.00	18.00	15.00	10.00	4.00	2.00
'C17U3N50'	75	1.00	0.00	1.00	3.00	2.00	6.00	19.00	27.00	27.00	21.00	13.00	8.00	3.00
'C17U3N60'	76	0.00	0.00	1.00	4.00	10.00	16.00	32.00	35.00	33.00	22.00	17.00	7.00	2.00
'C17U3N70'	77	10.00	6.00	6.00	9.00	13.00	20.00	30.00	35.00	27.00	19.00	17.00	5.00	1.00
'C19U1N50'	78	0.00	0.00	0.50	1.00	0.50	10.00	25.50	28.50	26.00	29.50	31.00	31.50	36.00
'C19U1N65'	79	0.00	0.00	0.00	2.00	0.50	3.00	2.50	14.50	14.00	11.50	7.50	7.00	6.00
'C19U2N20'	80	0.00	0.00	0.00	0.00	3.00	15.00	21.00	21.00	22.00	20.00	27.00	25.00	24.00
'C19U2N40'	81	4.50	2.00	7.00	9.00	12.50	19.50	24.00	26.50	29.00	27.50	30.50	27.00	32.00
'C19U2N60'	82	3.00	7.00	5.00	5.00	13.00	20.00	30.00	37.00	36.00	38.00	35.00	40.00	34.00
'C19U3N40'	83	0.00	0.00	0.00	0.00	0.00	0.00	1.00	0.00	1.00	2.00	1.00	1.00	1.00
'C19U3N60'	84	0.00	0.00	0.00	0.00	0.00	0.00	0.33	0.33	1.67	7.67	11.33	11.33	12.33
'C19U3S40'	85	1.00	2.00	0.00	0.00	0.00	0.00	0.00	0.00	0.00	0.00	1.00	33.00	82.00
'C19U3S60'	86	3.33	6.33	5.33	31.33	76.67	80.00	97.00	81.67	110.33	97.67	81.33	83.00	87.00
'C19U5N20'	87	0.33	1.00	0.67	0.67	4.00	12.00	15.67	17.00	19.33	19.33	19.67	20.00	20.00
'C19U5N40'	88	3.00	8.00	12.00	13.00	20.00	20.00	20.00	20.00	20.00	20.00	20.00	21.00	20.00
'C19U5N60'	89	3.00	5.00	13.00	13.00	19.00	20.00	20.00	20.00	20.00	20.00	23.00	20.00	20.00
'C31U2N50'	90	0.00	0.00	0.00	0.00	0.00	0.00	11.00	14.00	19.00	20.00	20.00	20.00	21.00
'C31U2N60'	91	0.00	0.00	0.00	0.00	7.00	12.00	16.00	15.00	17.00	18.00	21.00	17.00	16.00
'C31U2N70'	92	0.00	1.00	3.00	4.00	9.00	11.00	10.00	14.00	12.00	16.00	19.00	20.00	21.00
'C31U2S50'	93	0.00	0.00	0.00	0.00	0.00	0.00	0.00	0.00	1.00	5.00	5.00	14.00	15.00
'C31U2S60'	94	0.00	0.00	0.00	0.00	0.00	1.00	1.00	3.00	9.00	20.00	22.00	23.00	23.00
'C31U2S70'	95	0.00	0.00	0.00	0.00	0.00	0.00	4.00	3.00	9.00	21.00	19.00	22.00	21.00
'C36U1N50'	96	0.00	1.00	1.00	10.00	19.00	25.00	34.00	23.00	14.00	6.00	3.00	3.00	2.00
'C36U2N50'	97	14.00	19.00	21.00	21.00	21.00	22.00	22.00	20.00	22.00	23.00	19.00	20.00	21.00
'C36U2S50'	98	13.00	24.00	38.00	50.00	74.00	87.00	89.00	94.00	100.00	92.00	93.00	93.00	108.00
'C36U4N30'	99	7.33	11.67	13.00	17.00	17.33	22.00	30.00	32.00	29.33	29.00	30.67	29.33	27.00
'C36U4N40'	100	3.67	2.33	5.00	12.67	14.67	22.00	31.67	30.00	25.00	20.33	22.00	20.33	16.00
'C36U4S30'	101	7.67	13.67	18.67	26.00	37.33	48.67	62.33	50.33	46.33	45.33	46.67	38.67	21.33
'C36U4S40'	102	4.00	4.33	9.67	19.67	27.33	26.67	23.67	15.33	5.67	6.67	4.00	7.67	12.67
'C36U5N30'	103	0.00	0.00	0.00	0.00	0.00	0.00	0.00	2.00	8.00	17.00	28.00	23.00	23.00
'C36U5N50'	104	0.00	0.00	0.00	0.00	0.00	0.00	0.00	0.00	0.00	6.00	11.00	19.00	14.00
'C36U5N70'	105	0.00	0.00	0.00	0.00	0.00	0.00	0.00	0.00	0.00	0.00	2.00	3.00	9.00
'C36U6N40'	106	20.00	22.00	24.00	22.00	18.00	20.00	17.00	19.00	16.00	21.00	18.00	13.00	13.00
'C36U6S40'	107	102.00	101.00	109.00	114.00	119.00	120.00	135.00	119.00	108.00	109.00	102.00	98.00	91.00
'C36U7N30'	108	35.00	48.00	45.00	45.00	42.00	36.00	39.00	36.00	38.00	37.00	35.00	28.00	29.00
'C36U7S30'	109	34.00	40.00	45.00	49.00	63.00	64.00	49.00	59.00	55.00	47.00	55.00	43.00	46.00
'C36U8N50'	110	0.00	0.00	0.00	0.00	2.50	5.00	10.50	10.00	5.50	6.00	4.50	6.00	10.50
'C47U1N55'	111	0.00	0.00	0.00	1.00	1.00	5.00	30.00	31.00	26.00	22.00	19.00	16.00	9.00
'C47U1N65'	112	0.00	0.00	0.00	0.00	1.00	3.00	31.00	37.00	29.00	19.00	16.00	9.00	14.00
'C47U1N80'	113	0.00	0.00	0.00	1.00	1.00	12.00	37.00	31.00	18.00	14.00	10.00	10.00	15.00
'C47U2N55'	114	4.00	11.00	10.00	17.00	14.00	24.00	7.00	2.00	2.00	3.00	2.00	3.00	3.00
'C47U2S55'	115	43.00	62.00	63.00	91.00	96.00	78.00	28.00	57.00	46.00	71.00	49.00	32.00	20.00
'C47U3N40'	116	5.00	12.00	16.00	13.00	3.00	5.00	5.00	7.00	5.00	2.00	6.00	11.00	3.00
'C47U3N50'	117	4.00	8.00	10.00	7.00	16.00	16.00	11.00	4.00	8.00	2.00	1.00	4.00	4.00
'C47U3N60'	118	2.00	7.00	4.00	4.00	9.00	1.00	1.00	1.00	1.00	4.00	1.00	3.00	3.00
'C47U3N70'	119	18.00	19.00	12.00	11.00	4.00	3.00	5.00	4.00	4.00	3.00	4.00	2.00	1.00
'C47U3S40'	120	29.00	43.00	53.00	54.00	42.00	30.00	43.00	38.00	40.00	51.00	68.00	64.00	63.00
'C47U3S50'	121	25.00	37.00	47.00	63.00	48.00	46.00	33.00	36.00	27.00	20.00	12.00	15.00	11.00
'C47U3S60'	122	29.00	37.00	45.00	50.00	34.00	19.00	9.00	3.00	4.00	13.00	7.00	9.00	5.00
'C47U3S70'	123	51.00	52.00	26.00	22.00	13.00	13.00	6.00	6.00	12.00	20.00	13.00	21.00	17.00
'C47U4N30'	124	110.00	110.00	103.00	111.00	113.00	116.00	123.00	109.00	115.00	118.00	113.00	116.00	125.00
'C47U4N40'	125	116.00	111.00	104.00	94.00	95.00	91.00	95.00	94.00	100.00	117.00	117.00	132.00	138.00
'C47U4N50'	126	155.00	158.00	151.00	135.00	131.00	131.00	138.00	152.00	171.00	175.00	188.00	188.00	186.00
'C47U4N60'	127	136.00	128.00	134.00	122.00	126.00	99.00	95.00	109.00	139.00	137.00	143.00	151.00	150.00
'C47U4N70'	128	131.00	130.00	136.00	144.00	132.00	134.00	149.00	143.00	124.00	126.00	127.00	123.00	117.00
'C47U5N30'	129	0.00	0.00	0.00	0.00	0.00	0.00	17.00	35.00	56.00	62.00	61.00	65.00	63.00
'C47U5N50'	130	0.00	0.00	0.00	0.00	0.00	0.00	8.00	42.00	49.00	60.00	64.00	71.00	67.00
'C47U5N70'	131	0.00	0.00	0.00	0.00	0.00	0.00	18.00	45.00	52.00	55.00	53.00	54.00	50.00
'C47U6N70'	132	0.00	0.00	0.00	1.00	4.00	9.00	25.00	39.00	38.00	32.00	30.00	19.00	15.00
'C47U6N80'	133	0.00	0.00	0.00	0.00	0.00	5.00	37.00	30.00	9.00	7.00	3.00	0.00	0.00

'C47U7N50'	134	0.00	0.00	0.00	0.00	0.00	0.00	5.00	8.00	23.00	25.00	24.00	20.00	28.00
'C47U7N60'	135	0.00	0.00	0.00	0.00	0.00	2.00	15.00	32.00	35.00	37.00	35.00	36.00	36.00
'C47U7N70'	136	0.00	0.00	0.00	0.00	0.00	0.00	25.00	37.00	41.00	42.00	40.00	40.00	38.00
'C47U7N80'	137	0.00	0.00	0.00	0.00	1.00	7.00	21.00	37.00	34.00	34.00	39.00	42.00	39.00
'C49U1N50'	138	0.00	0.00	0.00	1.00	7.00	15.00	20.00	16.00	14.00	8.00	3.00	2.00	0.00
'C49U1N60'	139	0.00	0.00	0.00	1.00	21.00	23.00	21.00	16.00	13.00	2.00	0.00	0.00	0.00
'C49U1N64'	140	0.00	0.00	0.00	0.00	0.00	4.00	10.00	20.00	37.00	35.00	31.00	23.00	16.00
'C49U1N73'	141	0.00	0.00	0.00	0.00	1.00	21.00	83.00	99.00	91.00	72.00	52.00	38.00	25.00
'C49U1N74'	142	0.00	0.00	0.00	0.00	2.00	19.00	37.00	42.00	36.00	27.00	20.00	19.00	11.00
'C49U1N75'	143	0.00	0.00	0.00	12.00	20.00	23.00	20.00	16.00	6.00	3.00	1.00	1.00	0.00
'C49U2N40'	144	8.00	9.00	20.00	23.00	28.00	32.00	29.00	34.00	33.00	31.00	28.00	20.00	14.00
'C49U2N42'	145	5.00	6.00	7.00	7.00	11.00	18.00	19.00	17.00	13.00	7.00	5.00	3.00	4.00
'C49U2N43'	146	11.00	16.00	19.00	21.00	20.00	20.00	21.00	21.00	20.00	15.00	10.00	7.00	1.00
'C49U2N60'	147	20.00	20.00	19.00	19.00	20.00	20.00	24.00	22.00	19.00	24.00	15.00	13.00	11.00
'C49U2N62'	148	11.00	11.00	11.00	17.00	18.00	17.00	20.00	19.00	16.00	7.00	8.00	2.00	0.00
'C49U2N63'	149	15.00	18.00	18.00	20.00	20.00	20.00	21.00	22.00	23.00	18.00	16.00	12.00	8.00
'C49U2N80'	150	16.00	9.00	11.00	14.00	15.00	13.00	13.00	13.00	10.00	5.00	2.00	3.00	1.00
'C49U2N81'	151	3.00	2.00	12.00	12.00	9.00	15.00	13.00	10.00	5.00	6.00	0.00	0.00	0.00
'C49U2N82'	152	3.00	1.00	6.00	4.00	6.00	9.00	10.00	10.00	4.00	2.00	1.00	1.00	0.00
'C49U2N83'	153	7.00	9.00	9.00	8.00	13.00	17.00	8.00	10.00	10.00	6.00	4.00	0.00	1.00
'C49U3N25'	154	23.00	22.00	20.00	26.00	29.00	27.00	40.00	42.00	39.00	40.00	39.00	38.00	36.00
'C49U3N40'	155	18.00	20.00	31.00	36.00	37.00	38.00	38.00	39.00	34.00	31.00	24.00	25.00	21.00
'C49U3N42'	156	10.00	13.00	15.00	12.00	11.00	15.00	20.00	20.00	26.00	30.00	25.00	27.00	28.00
'C49U3N43'	157	19.00	17.00	17.00	18.00	19.00	20.00	20.00	20.00	24.00	23.00	21.00	20.00	20.00
'C49U3N60'	158	28.00	26.00	32.00	31.00	24.00	22.00	21.00	21.00	21.00	20.00	22.00	20.00	20.00
'C49U3N61'	159	11.00	7.00	4.00	2.00	11.00	21.00	20.00	20.00	18.00	20.00	19.00	20.00	19.00
'C49U3N62'	160	16.00	21.00	22.00	22.00	22.00	21.00	22.00	21.00	21.00	19.00	20.00	20.00	20.00
'C49U3N63'	161	21.00	21.00	22.00	21.00	21.00	20.00	22.00	21.00	21.00	22.00	20.00	21.00	20.00
'C49U3N70'	162	22.00	25.00	21.00	20.00	20.00	19.00	20.00	20.00	20.00	20.00	20.00	20.00	19.00
'C49U3N80'	163	5.00	2.00	8.00	13.00	19.00	20.00	21.00	20.00	20.00	20.00	19.00	20.00	20.00
'C49U3N81'	164	20.00	21.00	21.00	20.00	20.00	20.00	19.00	21.00	17.00	19.00	18.00	19.00	16.00
'C49U3N82'	165	22.00	20.00	21.00	19.00	19.00	19.00	20.00	20.00	20.00	20.00	20.00	16.00	16.00
'C49U3N83'	166	20.00	21.00	20.00	21.00	21.00	21.00	20.00	20.00	20.00	20.00	21.00	20.00	18.00
'C49U3N84'	167	1.00	0.00	0.00	3.00	4.00	10.00	17.00	17.00	17.00	16.00	19.00	17.00	13.00
'C49U4N41'	168	0.00	0.00	0.00	0.00	0.00	1.00	2.00	0.00	5.00	5.00	10.00	2.00	2.00
'C49U4N43'	169	0.00	0.00	1.00	0.00	0.00	0.00	3.00	3.00	4.00	3.00	9.00	4.00	9.00
'C49U4N44'	170	0.00	1.00	0.00	1.00	0.00	0.00	2.00	1.00	0.00	3.00	5.00	5.00	8.00
'C49U4N51'	171	0.00	0.00	0.00	0.00	5.00	5.00	7.00	9.00	9.00	11.00	9.00	7.00	3.00
'C49U4N52'	172	0.00	0.00	0.00	0.00	1.00	0.00	2.00	3.00	16.00	12.00	13.00	14.00	7.00
'C49U4N53'	173	0.00	0.00	2.00	2.00	3.00	3.00	5.00	5.00	4.00	8.00	14.00	15.00	19.00
'C49U4N54'	174	0.00	0.00	1.00	1.00	1.00	1.00	5.00	11.00	16.00	18.00	34.00	16.00	16.00
'C49U4N60'	175	0.00	0.00	0.00	0.00	0.00	0.00	7.00	18.00	25.00	28.00	23.00	13.00	15.00
'C49U4N61'	176	0.00	0.00	0.00	0.00	0.00	1.00	3.00	6.00	4.00	3.00	5.00	2.00	0.00
'C49U4N62'	177	0.00	0.00	0.00	0.00	0.00	0.00	4.00	11.00	25.00	26.00	10.00	9.00	4.00
'C49U4N63'	178	0.00	0.00	0.00	0.00	0.00	0.00	4.00	3.00	13.00	17.00	11.00	8.00	4.00
'C49U4N64'	179	0.00	0.00	0.00	0.00	1.00	2.00	2.00	6.00	11.00	19.00	23.00	25.00	4.00
'C49U4N70'	180	0.00	0.00	0.00	0.00	0.00	2.00	2.00	7.00	16.00	3.00	3.00	1.00	0.00
'C49U4N73'	181	1.00	0.00	2.00	0.00	1.00	1.00	0.00	3.00	1.00	4.00	4.00	4.00	7.00
'C49U4N74'	182	2.00	5.00	3.00	2.00	0.00	2.00	4.00	17.00	10.00	17.00	21.00	16.00	15.00
'D09U1N20'	183	0.00	2.00	0.00	1.00	9.00	12.00	13.00	10.00	10.00	10.00	14.00	11.00	15.00
'D09U1N30'	184	0.00	1.00	4.00	6.00	12.00	18.00	23.00	27.00	22.00	9.00	17.00	13.00	7.00
'D09U1N40'	185	3.00	1.00	12.00	6.00	11.00	15.00	20.00	21.00	19.00	15.00	13.00	3.00	6.00
'D09U1N50'	186	3.00	2.00	1.00	4.00	3.00	7.00	14.00	21.00	14.00	10.00	8.00	4.00	5.00
'D09U1N60'	187	1.00	0.00	2.00	0.00	0.00	4.00	14.00	16.00	15.00	12.00	5.00	5.00	8.00
'D09U1N70'	188	0.00	1.00	1.00	0.00	1.00	2.00	17.00	17.00	17.00	19.00	10.00	16.00	16.00
'D09U1N80'	189	0.00	0.00	0.00	0.00	0.00	3.00	11.00	16.00	17.00	20.00	21.00	20.00	22.00
'D09U2N30'	190	2.00	2.00	3.00	0.00	3.00	2.00	7.00	5.00	3.00	5.00	8.00	5.00	3.00
'D09U2N40'	191	0.00	1.00	1.00	1.00	3.00	6.00	19.00	12.00	3.00	9.00	4.00	2.00	2.00
'D09U2N50'	192	0.00	0.00	0.00	0.00	0.00	0.00	9.00	3.00	4.00	4.00	4.00	6.00	7.00
'D09U2N60'	193	0.00	0.00	1.00	0.00	1.00	3.00	6.00	2.00	6.00	6.00	5.00	9.00	9.00
'D09U2N70'	194	1.00	0.00	0.00	0.00	0.00	3.00	11.00	11.00	12.00	14.00	14.00	13.00	15.00
'D09U2N80'	195	0.00	0.00	1.00	1.00	4.00	8.00	21.00	13.00	11.00	13.00	16.00	14.00	14.00
'D15U3N50'	196	0.00	0.00	0.00	0.00	0.00	0.00	0.00	0.00	0.00	4.00	2.00	2.00	12.00
'D15U3N55'	197	0.00	0.00	0.00	0.00	0.00	0.00	4.00	2.00	8.00	4.00	14.00	12.00	12.00
'D15U3N60'	198	0.00	0.00	0.00	0.00	0.00	0.00	30.00	32.00	36.00	22.00	22.00	14.00	30.00
'D15U3N65'	199	0.00	0.00	0.00	0.00	0.00	12.00	34.00	44.00	30.00	24.00	28.00	24.00	34.00
'D15U3N70'	200	0.00	0.00	0.00	0.00	0.00	8.00	40.00	40.00	36.00	44.00	40.00	40.00	50.00
'D15U3N75'	201	0.00	0.00	0.00	0.00	0.00	2.00	38.00	50.00	36.00	30.00	36.00	44.00	60.00
'D15U3N80'	202	0.00	0.00	0.00	0.00	0.00	0.00	38.00	62.00	58.00	50.00	50.00	48.00	50.00
'D15U4N20'	203	0.00	0.00	0.00	0.00	0.00	0.00	0.00	0.00	0.00	0.00	6.00	26.00	26.00
'D15U4N30'	204	0.00	0.00	0.00	0.00	0.00	0.00	2.00	24.00	27.00	22.00	25.00	19.00	13.00
'D15U4N40'	205	0.00	0.00	0.00	6.00	31.00	28.00	26.00	23.00	10.00	13.00	11.00	4.00	2.00
'D15U4N50'	206	1.00	15.50	35.50	33.50	32.50	29.00	13.00	11.00	7.50	6.00	1.00	2.00	0.00
'D15U4N60'	207	17.00	13.00	11.50	11.50	3.00	6.50	2.00	3.00	0.50	1.50	0.00	0.00	1.00
'D15U4N70'	208	2.00	0.00	3.00	0.00	0.00	3.00	1.00	1.00	1.00	0.00	0.00	0.00	0.00
